# Correction: Liu et al. Analysis of Gut Microbial Communities and Functions in *Passer ammodendri* Under Two Extreme Environments. *Microorganisms* 2025, *13*, 2642

**DOI:** 10.3390/microorganisms14051164

**Published:** 2026-05-21

**Authors:** Yaqi Liu, Peng He, Dongxin Liu, Yang Song, Chenxi Jia, Duochun Wang, Qinghua Jin, Gang Song, Qiang Wei

**Affiliations:** 1 School of Medicine, Yanbian University, Yanji 133000, China; yqliu751@163.com (Y.L.); yqinghua@ybu.edu.cn (Q.J.); 2 National Pathogen Resource Center, Chinese Center for Disease Control and Prevention (Chinese Academy of Preventive Medicine), Beijing 102206, China; liucommon@126.com; 3 National Animal Collection Resource Center, Institute of Zoology, Chinese Academy of Sciences, Beijing 100101, China; hepeng@ioz.ac.cn; 4 Division of Infectious Disease, Chinese Center for Disease Control and Prevention (Chinese Academy of Preventive Medicine), Beijing 102206, China; songyang@chinacdc.cn; 5 Key Laboratory of Animal Biodiversity Conservation and Integrated Pest Management, Institute of Zoology, Chinese Academy of Sciences, Beijing 100101, China; jiacx@ioz.ac.cn; 6 National Institute for Communicable Disease Control and Prevention, Chinese Center for Disease Control and Prevention (China CDC), Beijing 102206, China; wangduochun@icdc.cn

In the original publication [1], there was a mistake in the legend for Table 1: “The relative abundances of bacteria with a relative frequency of more than 0.1% at the species level of the two groups.” During the article revision process, the description of “Dominant Bacteria” in the text was replaced with “species with a relative abundance > (or more than) 0.1%”. However, this modification was overlooked during the revision process. Furthermore, due to our oversight, there were also errors in the translation of the title of Table 1. To maintain consistency with the content of the text, we would like to change the original “a relative frequency” to “relative abundances”. The correct legend appears below. 

**Table 1:** The relative abundances of bacteria with relative abundances of more than 0.1% at the species level of the two groups.

	**Relative Abundance (%)**	**Pamir Plateau Group (M)**	**Tazhong Town Group (FKDN)**
**Species**			

**Text Correction**

There were some errors in the original publication [1].

(1) In Section 3.2, “Composition and Relative Abundance of Gut Microbiota,” due to our oversight, there were errors in the description of the composition of bacterial species at the genus and species levels, and there was also an error in the Latin name of one species. To avoid any misunderstanding, we request that the following corrections be made. The revised content is excerpted as follows:

“the family level (Figure 3D, Table S7) includes *Clostridiaceae*, *Enterobacteriaceae*, *Enterococcaceae*, *Mycoplasmataceae*, and Comamonadaceae; the genus level (Figure 3E, Table S8) includes *Candidatus Arthromitus*, *Escherichia*, *Shigella*, *Enterococcus*, *Mycoplasma*, and *Klebsiella*; the species level (Figure 3F, Table S9) includes *Candidatus Arthromitus* sp. *SFB rat Yit*, *Escherichia coli*, *Enterococcus faecium*, *Enterococcus faecalis*, and *Klebsiella pneumoniae*.” 

(2) In Section 3.2, “Composition and Relative Abundance of Gut Microbiota”, in the paragraph above Table 1, the term “dominant bacteria” in the first sentence was changed to “bacteria with a relative frequency of more than 0.1%”.

(3) There are two instances in the Abstract where the translation of “Tazhong Town group” is not quite appropriate. The original text states “Tazhong group”, and to maintain consistency throughout the document, we suggest modifying it to “Tazhong Town group”. The revised excerpt is as follows:

“In addition, *Lysinibacillus sphaericus* is a unique strain specific to the Tazhong Town group, while *Stenotrophomonas maltophilia* has a much higher abundance in the Tazhong Town group than in the Pamir Plateau group.”

(4) In Section 3.4, “Functional Prediction of Gut Microbiota in Two Groups of Samples,” the translation of “mobile elements” in the figure caption differs from that in Figure 6E. To maintain consistency, we suggest replacing “mobile genetic elements” in the caption with “mobile elements”. The revised content is excerpted as follows:

“(**E**) Relative abundance of bacteria containing mobile elements OTUs. The horizontal axis represents the groups, and the vertical axis represents the relative abundance percentage (displayed as a decimal).”

(5) In the Discussion section, to avoid ambiguity, we hope to add “respectively” to the sentence to improve reader understanding. The revised content is excerpted as follows:

“The Pamir Plateau and Tazhong Town are respectively located in the southwest of Xinjiang and the central part of the Tarim Basin.”

(6) In the Acknowledgements section, we found one sentence to be somewhat redundant, and we hope to delete it. The revised content is excerpted as follows:

“Acknowledgments: We would like to express our gratitude to ANNOROAD GENE TECHNOLOGY for the technical support provided for this research. During the process of translating the article, the authors used NetEase Youdao APP (11.2.9) for the purposes of auxiliary translation and polishing. The authors have reviewed and edited the output and take full responsibility for the content of this publication.”

(7) The “Saxaul sparrow” keyword was non-italicized and in lowercase.

The authors state that the scientific conclusions are unaffected. These corrections were approved by the Academic Editor. The original publication has also been updated.
