# Supplementary material for: Analysis of Gut Microbial Communities and Functions in Passer ammodendri Under Two Extreme Environments
Source: Microorganisms. 2025 Nov 20;13(11):2642. doi: 10.3390/microorganisms13112642 (PMC12655348; doi:10.3390/microorganisms13112642)
Supplement: Supplementary file 1 [file microorganisms-13-02642-s001.zip › microorganisms-3931826-supplementary.pdf]

Supplementary Files

Supplementary Tables

Table S1. Sample sequencing data processing and OTU annotation result statistics

Sample ID	Raw CCS	Clean CCS	Effective CCS	AvgLen(bp)	Effective(%)	OTU Num	Seqs Num
FKDN1	17802	17802	16340	1454	91.79	99	14134
FKDN10	17810	17810	15978	1462	89.71	123	13688
FKDN11	16006	16006	15981	1438	99.84	54	15782
FKDN2	18353	18353	16396	1458	89.34	125	14537
FKDN3	17999	17999	16180	1471	89.89	121	14822
FKDN4	17711	17711	17163	1463	96.91	86	16345
FKDN5	17104	17104	16502	1459	96.48	116	15139
FKDN6	18711	18711	17507	1456	93.57	52	16554
FKDN7	11724	11724	10678	1455	91.08	153	8694
FKDN8	17786	17786	16856	1441	94.77	76	15765
FKDN9	18057	18055	15732	1454	87.12	82	14400
FKDNs1	17111	17111	15882	1451	92.82	210	8748
FKDNs3	18672	18672	18426	1427	98.68	50	17767
FKDNs4	17474	17473	15161	1462	86.76	102	13933
M24008	58075	58047	57709	1464	99.37	119	55823
M24015	55151	55142	55081	1448	99.87	87	54387
M24016	51056	51023	50648	1443	99.2	141	48589

Sample ID	Raw CCS	Clean CCS	Effective CCS	AvgLen(bp)	Effective(%)	OTU Num	Seqs Num
M24026	63672	63660	62991	1471	98.93	59	62416
M24033	62039	62020	61866	1441	99.72	72	60410
M24039	54889	54860	54470	1467	99.24	97	53288
Average	FKDN：17308.57<br>M：57480.33	FKDN：17308.36<br>M：57458.67	FKDN：16055.85714<br>M：57127.50	1454.25	94.7545	—	—
Total	—	—	—	—	—	526	535221

Sample ID represents the sample name; Raw CCS indicates the number of CCS identified for this sample; Clean CCS is the number of sequences after primer identification and removal; Effective CCS is the number of sequences used for subsequent analysis after length filtering and removal of chimeras; AvgLen (bp) represents the average sequence length of the sample; Effective (%) is the percentage of Effective CCS relative to Raw CCS. CCS: Circular Consensus Sequencing.

Table S2. Alpha Diversity Index Statistics

Sample ID	ACE	Shannon	Coverage
FKDN1	115.4758	3.1016	0.9986
FKDN10	207.0454	4.5368	0.9981
FKDN11	152.5801	1.2507	0.9983
FKDN2	139.7001	3.6539	0.9987
FKDN3	191.3735	4.2144	0.9978
FKDN4	132.1946	1.6764	0.9983
FKDN5	217.96	3.3707	0.9974
FKDN6	70.3564	2.012	0.9991
FKDN7	250.8449	4.8072	0.9946
FKDN8	96.7774	1.9072	0.9984
FKDN9	107.2435	3.8206	0.999
FKDNs1	252.0173	5.2474	0.9944
FKDNs3	104.3702	1.6271	0.999
FKDNs4	110.7829	4.3354	0.9993
M24008	136.4887	0.4431	0.9996
M24015	104.1857	2.4141	0.9997
M24016	167.1212	0.7622	0.9993
M24026	84.3099	1.7016	0.9996
M24033	130.0919	0.6932	0.9996

Sample ID	ACE	Shannon	Coverage
M24039	120.257	2.3909	0.9995

Sample ID represents Sample Name; Feature represents Number of features (OTUs); ACE represents Abundance-based Coverage Estimator - an index used to estimate the number of species in the community based on abundance; Chao1 represents Chao1 index - one of the measures for species richness; Simpson represents Simpson index - used to estimate one of the diversity indices of microorganisms in the sample; Shannon represents Shannon-Wiener index - used to estimate one of the diversity indices of microorganisms in the sample; PD whole tree represents Diversity index calculated based on the phylogenetic tree - the larger the value, the higher the community diversity; Coverage represents Coverage of the sample library;

Table S3: Statistical Table of Species at Each Classification Level of the Samples

Sample	Phylum	Class	Order	Family	Genus	Species
FKDN1	8	9	22	39	56	73
FKDN10	13	17	34	47	64	79
FKDN11	9	12	19	30	36	39
FKDN2	11	15	31	47	60	84
FKDN3	13	17	32	49	63	74
FKDN4	10	13	24	42	50	59
FKDN5	13	16	31	50	66	80
FKDN6	8	11	19	27	32	36
FKDN7	16	19	38	59	83	103
FKDN8	7	8	18	32	47	55
FKDN9	12	14	27	35	42	49
FKDNs1	17	21	42	71	111	152
FKDNs3	3	6	14	20	28	35
FKDNs4	12	15	31	44	49	58
M24008	8	11	32	57	83	98
M24015	9	13	34	52	70	78
M24016	8	11	33	64	98	119
M24026	7	8	24	35	46	51
M24033	6	9	22	36	51	61

Sample	Phylum	Class	Order	Family	Genus	Species
M24039	7	10	29	50	69	84
Total	22	32	73	129	241	340

The "Sample" column represents the sample names; Kingdom, Phylum, Class, Order, Family, Genus, and Species respectively denote the number of species annotated at each classification level.

Table S4: Annotation Results of Species at the Phylum Level

Phylum	M24008	M24015	M24016	M24026	M24033	M24039
Firmicutes	0.02991598445085359	0.8899369334583632	0.944781740723209	0.7899577031530377	0.9982287700711803	0.46085422609217835
Proteobacteria	0.961969080844813	0.10414253406144851	0.04511309144044948	0.00038451679056652143	0.0008938917397781825	0.5380010508932592
Nanoarchaeota	0	0.00029418794932612575	0	0	0	0
Spirochaetota	0	0.00016548072149594572	0	0	0.00018208905810296309	0.00015012760846719713
Campylobacterota	3.5827526288447414e-05	0	0.0027784066352466606	0.20940143552935145	0	0.00022519141270079568
unclassified Archaea	0	0.00044128192398918857	0	0	0	0
unclassified Bacteria	0	0	0	0	0	0
Actinobacteriota	0.0018630313669992656	0.0045047529740563	0.003313507172405277	0.00016021532940271724	0.0005628207250455222	0.0005442125806935895
Bacteroidota	0.004711319706930835	0.0002758012024932429	0.003334087962295993	4.806459882081518e-05	0.00011587485515643106	0.00015012760846719713
Deinococcota	0	0	0	0	0	0
Others	0.00017913763144223707	0.00023902770882747713	0.00018522710901644406	4.806459882081518e-05	1.6553550736633007e-05	7.506380423359856e-05

Phylum	M24008	M24015	M24016	M24026	M24033	M24039
Unassigned	0.0013256184726725543	0	0.0004939389573771841	0	0	0

Phylum	FKDN3	FKDN1	FKDN2	FKDN4	FKDN5	FKDN6	FKDN7	FKDN8	FKDN9	FKDN10	FKDN11	FKDNs1	FKDNs3	FKDNs4
Firmicutes	0.029550667926055863	0.6399462289514646	0.599229552177203	0.945610278372591	0.07193341700244402	0.03763440860215054	0.4324821716126064	0.12331113225499524	0.11368055555555556	0.4179573348918761	0.002534533012292485	0.529835390946502	0.02093769347667023	0.3615875977894208
Proteobacteria	0.000202401835109 97166	0	0.0002063699525349109 2	0	0.00059449104960 69754	0	0.0003450655624 568668	0	0.00027777777777778	0.01979836353009 9356	0.019642630845266 76	0.0005715592135345222	5.62841222491135 3e-05	7.1772051962965 62e-05
Nanoarchaeota	0.5559978410470922	0.0015565303523418707	0.04952878860837862	0.016396451514224534	0.0533720853424929	0.003443276549474447	0.04819415688980906	0.00038058991436726926	0.0011805555555555556	0.09709234365867914	0.002914712964136358	0.036236854138088706	0.0028142061124556764	0.01995263044570444
Spirochaetota	0	0.0712466393094 6654	0.005571988718442594	0.00042826552462526765	0.0009247638549441839	0.00012081672103419114	0.010121923165401427	0.4860767522993974	6.9444444444444444e-05	7.305669199298655e-05	0.5402357115701432	0.0014860539551897576	0.5055439860415377	0.0006459484676666906
Campylobacterota	0.1399946026177304	0.0506579878307627	0.11597991332461993	0.004772101560110126	0.03329149877799062	0.0037453183520599 25	0.33229813664596275	0.003171582619727244	0.005208333333333333	0.1032291057860 9	0.0009504498796096819	0.16860996799268405	0.0025327855012101086	0.07686786765233618
unclassified_Archaea	0.0010120	0.000495	6.87899	0.00036	0.000660	0.351999	0.014147	0	0.147986	0.000876	0.0005702	0.001714	0.405808	0.003875

Phylum	FKDN3	FKDN1	FKDN2	FKDN4	FKDN5	FKDN6	FKDN7	FKDN8	FKDN9	FKDN10	FKDN11	FKDNs1	FKDNs3	FKDNs4
	091755498584	2596575633224	8417830364e-05	70847353930866	5456106744171	51673311586	688060731538		11111111112	6803039158387	699277658091	6776406035665	5214161085	6908060001436
unclassified_Bacteria	0.009985157198758601	0.0002830055186076129	0.09692508770722982	0.001223615784643622	0.4296849197437083	6.040836051709557e-05	0.009086726478030827	0.00019029495718363463	0.09618055555555556	0.11550263004091174	0	0.010402377686328304	0.05144368773568976	0.006961889040407666
Actinobacteriota	0.0004722709485899339	0.020517900099051932	0.008873907959001169	0.008687672070969715	0.030451152652090627	0.002597559502235109	0.03347135955831608	0.0038693307960672376	0.051458333333333335	0.12456165984804209	0.0006336332530731213	0.031321444901691815	0.009005459559858164	0.33330940931601233
Bacteroidota	0.00026986911347996224	0.0003537568982595161	0	0.000611807892321811	0.00019816368320232512	0.44502839192944305	0.00540602714515758	0	0.18756944444444446	0.00021917007597895967	0.0004435432771511849	0.00205761316872428	0	0.002081389506926003
Deinococcota	0.00020240183510997166	0.06855808688269421	0.005915938639334113	0.00042826552462526765	0.00019816368320232512	0.0817929201401474	0.0011502185415228894	0	0.0599305555555555556	0.00021917007597895967	0.00012672665061462426	0.0013717421124828531	0	0.0005024043637407593
Others	0.26231277830252314	0.0858921748974105	0.11350347389420101	0.02067910676047721	0.37869079859964333	0.07345656638878824	0.11329652634000459	0.014145258483983507	0.33645833333333334	0.11988603156049099	0.001964263084526676	0.2147919524462735	0.0018573760342207462	0.19349745209215533
Unassigned	0	0.060492429602377246	0.004196189034876522	0.0007953502600183542	0	0.00012081672103419114	0	0.3688550586742785	0	0.0005844535359438924	0.4299835255354201	0.001600365797896662	0	0.0006459484676666906

Phylum represents each bacterial phylum; M24008, M24015, M24016, M24026, etc. represent the relative abundance of each bacterial phylum in each sample.

Table S5: Annotation Results of Species at the Class Level

Class	M24008	M24015	M24016	M24026	M24033	M24039
Gammaproteobacteria	0.9607688587141501	0.10011583650504716	0.04321965877050361	0.00038451679056652143	0.0008607846383049164	0.5379259870890257
Bacilli	0.024685165612740267	0.31503852023461487	0.024511720759842762	0.7584273263265829	0.14502565800364178	0.3335647800630536
Clostridia	0.005230818838113323	0.5738319819074411	0.9202700199633662	0.03153037682645476	0.8531037907631187	0.12719561627383275
Nanoarchaeia	0	0.00029418794932612575	0	0	0	0
Spirochaetia	0	0.00016548072149594572	0	0	0.00018208905810296309	0.00015012760846719713
Campylobacteria	3.5827526288447414e-05	0	0.0027784066352466606	0.20940143552935145	0	0.00022519141270079568
unclassified Archaea	0	0.00044128192398918857	0	0	0	0
unclassified Bacteria	0	0	0	0	0	0
Alphaproteobacteria	0.0012002221306629884	0.004026697556401346	0.0018934326699458724	0	3.3107101473266015e-05	7.50638042335985 6e-05
Actinobacteria	0.0013614459989610018	0.004210565024730175	0.0030459569038259688	0.0001602153294027172 4	0.0005628207250455222	0.0005442125806935895
Others	0.00539204270641	0.001875448176954	0.003786865339891	9.612919764163036e-0	0.00023174971031286	0.00031902116799

Class	M24008	M24015	M24016	M24026	M24033	M24039
	1336	0515	745	5	21	27939
Unassigned	0.0013256184726725543	0	0.0004939389573771841	0	0	0

Class	FKDN3	FKDN1	FKDN2	FKDN4	FKDN5	FKDN6	FKDN7	FKDN8	FKDN9	FKDN10	FKDN11	FKDNs1	FKDNs3	FKDNs4
Gammaproteobacteria	0.18803130481716368	0.7665911985283713	0.7286235124165922	0.9610278372591007	0.13594028667679503	0.04584994563247553	0.8066482631700023	0.13295274341896607	0.17076388888888888	0.6481589713617767	0.00500570269927765 8	0.7622313671696388	0.0324759385377385	0.778726763798177
Bacilli	0.6461341249493996	0.07329842931937172	0.15670358395817569	0.03291526460691343	0.8443754541251073	0.003624501631025734	0.0840809753853232	0.4866476371709483	0.09986111111111111111	0.2363383985973115	0.5431504245342795	0.05829903978052126	0.5599707322564305	0.04492930452881648
Clostridia	0.041020105248954254	0	0.004402558987411433	6.11807892321811e-05	0.0023119096373604596	6.040836051709557e-05	0.017828387393604785	0	0.0009027777777777777	0.05837229690239626	0.019769357495881382	0.006630086877000457	0.00011256824449822705	0.02863704873323282
Nanoarchaeia	0.0010120091755498584	0.0004952596575633224	6.878998417830364e-05	0.0003670847353930866	0.0006605456106744171	0.35199951673311586	0.014147688060731538	0	0.14798611111111112	0.0008766803039158387	0.0005702699277658091	0.0017146776406035665	0.4058085214161085	0.0038756908060001436
Spirochaetia	0.00026986911347996224	0.0003537568982595161	0	0.000611807892321811	0.00019816368320232512	0.44502839192944305	0.00540602714515758	0	0.18756944444444446	0.00021917007597895967	0.0004435432771511849	0.00205761316872428	0	0.002081389506926003
Campylobacteria	0.000202401835109	0.0685580868826	0.005915938639	0.000428265524	0.00019816368320	0.0817929201401	0.00115021854152	0	0.0599305555555	0.00021917007597	0.0001267266506	0.00137174211248	0	0.0005024043637

Class	FKDN3	FKDN1	FKDN2	FKDN4	FKDN5	FKDN6	FKDN7	FKDN8	FKDN9	FKDN10	FKDN11	FKDNs1	FKDNs3	FKDNs4
	97166	9421	334113	62526765	232512	474	28894		55556	895967	1462426	28531		407593
unclassified Archaea	0.004250438537309405	0.00014150275930380644	0.020086675380064662	0.0003670847353930866	0.005218310324327895	0.06916757279207442	0.01943869335173683	0.0013954963526799874	0.130625	0.013296317942723554	0.0002534533012292485	0.006744398719707362	0	0.01636402784755616
unclassified Bacteria	0.00020240183510997166	0	0.0002063699525349109 2	0	0.00502014664112557	0	0.001610305958132045	0.0012686330478908975	0.16368055555555555	0.000657510227936879	0	0.00068587105624 14266	0	0
Alphaproteobacteria	0.006274456888409122	0.021862176312438093	0.0031643392722019675	0.0007953502600183542	0.002444018759495343	0.00012081672103419114	0.02035886818495514	0.00830954646368538	0.007569444444444445	0.004821741671537112	0.00012672665061462426	0.0720164609053498	0.0015196713007260651	0.015072130912222781
Actinobacteria	0.049386047766833086	0.0027593038064242254	0.0379032812822 4531	0.0013459773631079842	0.00171741858775 34844	0.0008457170472393379	0.004140786749482402	0.0004440215667618141 4	0.0015972222222222223	0.013442431326709527	0.00012672665061462426	0.030978509373571102	0	0.007536065456111391
Others	0.06321683983268116	0.005447856233196547	0.0387287610923 8495	0.0012847965738758027	0.0019155822709558095	0.0013893922918931981	0.02518978605935128	0.0001268633047890897 5	0.02951388888888888	0.023012857977790766	0.0004435432771511849	0.05566986739826245	0.00011256824449822705	0.10162922557955932
Unassigned	0	0.06049242960237 7246	0.00419618903487 6522	0.0007953502600183542	0	0.00012081672103419114	0	0.3688550586742785	0	0.0005844535359438924	0.4299835255354201	0.001600365797896662	0	0.0006459484676666906

Class represents each bacterial class; M24008, M24015, M24016, M24026, etc. represent the relative abundance of each bacterial class in each sample.

Table S6: Annotation Results of Species at the Order Level

Order	M24008	M24015	M24016	M24026	M24033	M24039
Enterobacterales	0.9597298604517851	0.08454226193759538	0.035110827553561506	0.00019225839528326071	0.00044694586988909123	0.5372879447530401
Clostridiales	0.0002507926840191319	0.5298692702300182	0.9158451501368623	0.031065752371186875	0.8508028472107267	0.125187659510584
Lactobacillales	0.020672482668434158	0.09783587989776969	0.020642532260388154	0.753476672648039	0.0018043370302929978	0.33088124906170246
Mycoplasmatales	0	0.1932998694540975	0	0.0043258138938733655	0.14234398278430724	0.0021205524695991594
Burkholderiales	0.0002507926840191319	0.011951385441373858	0.007717796209018502	8.010766470135862e-05	0.00013242840589306406	0.0005066806785767903
Woesearchaeales	0	0.00029418794932612575	0	0	0	0
Bacillales	0.0002687064471633556	5.516024049864857e-05	0.0005556813270493322	9.612919764163036e-05	0.00014898195662969708	0.00011259570635039784
Xanthomonadales	8.956881572111853e-05	0	6.174236967214802e-05	6.40861317610869e-05	1.6553550736633007e-05	1.876595105839964e-05
Spirochaetales	0	0.00016548072149594572	0	0	0.00018208905810296309	0.00015012760846719713
Campylobacterales	3.5827526288447414e-05	0	0.0027784066352466606	0.20940143552935145	0	0.00022519141270079568

Order	M24008	M24015	M24016	M24026	M24033	M24039
Others	0.017376350249897	0.08198650412782464	0.016793924550824263	0.00129774416816201	0.004121834133421619	0.0035092328479207325
Unassigned	0.0013256184726725543	0	0.0004939389573771841	0	0	0

Order	FKDN3	FKDN1	FKDN2	FKDN4	FKDN5	FKDN6	FKDN7	FKDN8	FKDN9	FKDN10	FKDN11	FKDNs1	FKDNs3	FKDNs4
Enterobacterales	0.029550667926055863	0.6399462289514646	0.599229552177203	0.945610278372591	0.07193341700244402	0.03763440860215054	0.4324821716126064	0.12331113225499524	0.11368055555555556	0.4179573348918761	0.002534533012292485	0.529835390946502	0.02093769347667023	0.3615875977894208
Clostridiales	0.00020240183510997166	0	0.00020636995253491092	0	0.0005944910496069754	0	0.0003450655624568668	0	0.000277777777777778	0.019798363530099356	0.01964263084526676	0.0005715592135345222	5.628412224911353e-05	7.177205196296562e-05
Lactobacillales	0.5559978410470922	0.0015565303523418707	0.04952878860837862	0.016396451514224534	0.0533720853424929	0.003443276549474447	0.04819415688980906	0.00038058991436726926	0.001180555555555556	0.09709234365867914	0.002914712964136358	0.036236854138088706	0.0028142061124556764	0.01995263044570444
Mycoplasmatales	0	0.07124663930946654	0.005571988718442594	0.00042826552462526765	0.0009247638549441839	0.00012081672103419114	0.010121923165401427	0.4860767522993974	6.94444444444444e-05	7.305669199298655e-05	0.5402357115701432	0.0014860539551897576	0.5055439860415377	0.0006459484676666906
Burkholderiales	0.139994602617730	0.0506579878307	0.115979913324	0.004772101560	0.0332914987779	0.0037453183520	0.3322981366459	0.00317158261972	0.0052083333333	0.1032291057860	0.00095044987960	0.168609967992	0.002532785501	0.0768678676523

Order	FKDN3	FKDN1	FKDN2	FKDN4	FKDN5	FKDN6	FKDN7	FKDN8	FKDN9	FKDN10	FKDN11	FKDNs1	FKDNs3	FKDNs4
	4	627	61993	110126	9062	59925	6275	7244	33333	9	96819	68405	2101086	3618
Woesearchaeales	0.0010120091755498584	0.0004952596575633224	6.878998417830364e-05	0.0003670847353930866	0.0006605456106744171	0.35199951673311586	0.014147688060731538	0	0.14798611111111112	0.0008766803039158387	0.0005702699277658091	0.0017146776406035665	0.4058085214161085	0.0038756908060001436
Bacillales	0.009985157198758601	0.0002830055186076129	0.09692508770722982	0.001223615784643622	0.4296849197437083	6.040836051709557e-05	0.009086726478030827	0.00019029495718363463	0.0961805555555556	0.11550263004091174	0	0.010402377686328304	0.05144368773568976	0.006961889040407666
Xanthomonadales	0.0004722709485899339	0.020517900099051932	0.008873907959001169	0.008687672070969715	0.030451152652090627	0.002597559502235109	0.03347135955831608	0.0038693307960672376	0.05145833333333335	0.12456165984804209	0.0006336332530731213	0.031321444901691815	0.009005459559858164	0.33330940931601233
Spirochaetales	0.00026986911347996224	0.0003537568982595161	0	0.000611807892321811	0.00019816368320232512	0.44502839192944305	0.00540602714515758	0	0.1875694444444446	0.00021917007597895967	0.0004435432771511849	0.00205761316872428	0	0.002081389506926003
Campylobacterales	0.00020240183510997166	0.06855808688269421	0.005915938639334113	0.00042826552462526765	0.00019816368320232512	0.0817929201401474	0.0011502185415228894	0	0.05993055555555556	0.00021917007597895967	0.00012672665061462426	0.0013717421124828531	0	0.0005024043637407593
Others	0.26231277830252314	0.0858921748974105	0.11350347389420101	0.02067910676047721	0.37869079859964333	0.07345656638878824	0.11329652634000459	0.014145258483983507	0.3364583333333334	0.11988603156049099	0.001964263084526676	0.2147919524462735	0.0018573760342207462	0.19349745209215533
Unassigned	0	0.0604924296023	0.004196189034	0.000795350260	0	0.0001208167210	0	0.36885505867427	0	0.0005844535359	0.42998352553542	0.001600365797	0	0.0006459484676

Order	FKDN3	FKDN1	FKDN2	FKDN4	FKDN5	FKDN6	FKDN7	FKDN8	FKDN9	FKDN10	FKDN11	FKDNs1	FKDNs3	FKDNs4
		77246	876522	0183542		3419114		85		438924	01	896662		666906

Order represents each bacterial order; M24008, M24015, M24016, M24026, etc. represent the relative abundance of each bacterial order in each sample.

Table S7: Annotation Results of Species at the Family Level

Family	M24008	M24015	M24016	M24026	M24033	M24039
Enterobacteriaceae	0.9591745337943142	0.08445032820343097	0.02514972524645496	0.00016021532940271724	0.0003972852176791922	0.5334033928839513
Clostridiaceae	0.0002507926840191319	0.5298692702300182	0.9158451501368623	0.0310657523711868 75	0.8508028472107267	0.125187659510584
Mycoplasmataceae	0	0.1932998694540975	0	0.0043258138938733655	0.14234398278430724	0.0021205524695991594
Enterococcaceae	8.956881572111853e-05	0.0026660782907680144	0.017082055609294284	0.7528678543963087	0.0006455884787286873	0.2333546014111995
unclassified Woesearchaeales	0	0.00029418794932612575	0	0	0	0
Comamonadaceae	3.5827526288447414e-05	0.01106882159339548	0.007429665150548478	0	0	1.876595105839964e-05
Planococcaceae	0	1.838674683288286e-05	6.174236967214802e-05	0	0	0
Xanthomonadaceae	8.956881572111853e-05	0	6.174236967214802e-05	6.40861317610869e-05	1.6553550736633007e-05	1.876595105839964e-05
Spirochaetaceae	0	0.00016548072149594572	0	0	0.00018208905810296309	0.00015012760846719713
Pseudoalteromonadaceae	0	0	0	0	0	0
Others	0.0390340898912	0.17816757681063	0.03387598016011	0.21151627787746724	0.0056116536997185	0.1057461342140

Family	M24008	M24015	M24016	M24026	M24033	M24039
	6345	49	8536		89	8197
Unassigned	0.0013256184726725543	0	0.0004939389573771841	0	0	0

Family	FKDN3	FKDN1	FKDN2	FKDN4	FKDN5	FKDN6	FKDN7	FKDN8	FKDN9	FKDN10	FKDN11	FKDNs1	FKDNs3	FKDNs4
Enterobacteriaceae	0.02901092969909594	0.6393094665345974	0.5912499140125198	0.9231569287243806	0.05918488671642777	0.03171438927147517	0.259949390384173	0.06045036473200127	0.10277777777777777	0.39874342489772063	0.0022177163857559244	0.19627343392775493	0.006022401080655147	0.26196798966482454
Clostridiaceae	0.00020240183510997166	0	0.00020636995253491092	0	0.0005944910496069754	0	0.0003450655624568668	0	0.000277777777777778	0.01979836353009356	0.01964263084526676	0.0005715592135345222	5.628412224911353e-05	7.177205196296562e-05
Mycoplasmataceae	0	0.07124663930946654	0.005571988718442594	0.00042826552462526765	0.0009247638549441839	0.00012081672103419114	0.010121923165401427	0.4860767522993974	6.944444444444444e-05	7.305669199298655e-05	0.5402357115701432	0.0014860539551897576	0.5055439860415377	0.0006459484676666906
Enterococcaceae	0.44467683173660777	0	0.004058609066519915	0.00893239522789844	0.0011889820992139507	0	0.005751092707614446	6.343165239454488e-05	0.000138888888888889	0.07524839275277616	0	0.011545496113397348	5.628412224911353e-05	0.003373286442259384
unclassified Woesearchaeales	0.00101200917554	0.0004952596575	6.878998417830	0.0003670847353	0.000660545610	0.3519995167331	0.01265240395	0	0.14798611111111	0.00087668030391	0.000570269927765	0.0016003657978	0.40580852141610	0.0017943012990

Family	FKDN3	FKDN1	FKDN2	FKDN4	FKDN5	FKDN6	FKDN7	FKDN8	FKDN9	FKDN10	FKDN11	FKDNs1	FKDNs3	FKDNs4
	98584	633224	364e-05	930866	6744171	1586	6751783		112	58387	8091	96662	85	741406
Comamonadaceae	0.1270408851706922	0.004528088297721806	0.10882575497007636	0.0020801468338941575	0.026884206354448773	0.0035640932705086384	0.3252818035426731	0.0019029495718363464	0.0024305555555555556	0.09753068381063705	0.0004435432771511849	0.133973479652492	0.0025327855012101086	0.057920045934113255
Planococcaceae	0.009310484415058697	0	0.0902524592419343 7	0.0007341694707861732	0.4220886452209525	6.040836051709557e-05	0.007936507936507936	0.0001268630478908975	0.09	0.007451782583284629	0	0.008573388203017833	0.04874204986773231	0.001363668987296 3468
Xanthomonadaceae	0.0004722709485899339	0.020517900099051932	0.008873907959001169	0.008687672070969715	0.030451152652090627	0.002597559502235109	0.03347135955831608	0.0038693307960672376	0.051458333333333335	0.12456165984804209	0.0006336332530731213	0.031321444901691815	0.009005459559858164	0.33330940931601233
Spirochaetaceae	0.00026986911347996224	0.0003537568982595161	0	0.000611807892321811	0.00019816368320232512	0.44502839192944305	0.00540602714515758	0	0.18756944444444446	0.00021917007597895967	0.0004435432771511849	0.00205761316872428	0	0.00208138950692 6003
Pseudoalteromonadaceae	0	0.00021225413895570964	0	0.0002447231569287244	0	6.040836051709557e-05	0.11088106740280654	0	0	0	6.336332530731213e-05	0.2781207133058985	0	0.00021531615588889685
Others	0.3880043179058154	0.20284420546200646	0.18669601705991612	0.053961456102783724	0.4578241627584385	0.16473359913011965	0.2282033586381413	0.07865524896923562	0.4172916666666667	0.2749123319696084	0.005766062602965403	0.3328760859625058	0.0222322282883 99845	0.3366109237063088

Family	FKDN3	FKDN1	FKDN2	FKDN4	FKDN5	FKDN6	FKDN7	FKDN8	FKDN9	FKDN10	FKDN11	FKDNs1	FKDNs3	FKDNs4
Unassigned	0	0.060492429602377246	0.004196189034876522	0.0007953502600183542	0	0.00012081672103419114	0	0.3688550586742785	0	0.0005844535359438924	0.4299835255354201	0.001600365797896662	0	0.0006459484676666906

Family represents each bacterial family; M24008, M24015, M24016, M24026, etc. represent the relative abundance of each bacterial family each sample.

Table S8: Annotation Results of Species at the Genus Level

Genus	M24008	M24015	M24016	M24026	M24033	M24039
Klebsiella	5.37412894326711 2e-05	1.83867468328828 6e-05	0.02455288233962 4195	4.80645988208151 8e-05	1.655355073663300 7e-05	0.06226542561 177
Candidatus Arthromitus	0	0.52933605457186 46	0.91549527670872 01	0.03096962317354 5246	0.850240026485681 2	0.05948806485 512686
Mycoplasma	0	0.19329986945409 75	0	0.00432581389387 33655	0.142343982784307 24	0.00212055246 95991594
Escherichia Shigella	0.95912079250488 15	0.08443194145659 809	0.00059684290683 07641	0.00011215073058 190208	0.000380731666942 55916	0.47113796727 21814
Enterococcus	8.95688157211185 3e-05	0.00266607829076 80144	0.01708205560929 4284	0.75286785439630 87	0.000645588478728 6873	0.23335460141 11995
unclassified Woesearchaeales	0	0.00029418794932 612575	0	0	0	0
Lysinibacillus	0	0	0	0	0	0
unclassified Spirochaetaceae	0	0	0	0	0.000165535507366 33007	0.00015012760 846719713
Stenotrophomonas	5.37412894326711 2e-05	0	4.11615797814320 1e-05	6.40861317610869 e-05	0	0
Delftia	0	0	0	0	0	0
Others	0.03935653762785 947	0.18995348153051 275	0.04173784189837 203	0.21161240707510 892	0.006207581526237 377	0.17148326077 165596

Genus	M24008	M24015	M24016	M24026	M24033	M24039
Unassigned	0.0013256184726725543	0	0.0004939389573771841	0	0	0

Genus	FKDN3	FKDN1	FKDN2	FKDN4	FKDN5	FKDN6	FKDN7	FKDN8	FKDN9	FKDN10	FKDN11	FKDNs1	FKDNs3	FKDNs4
Klebsiella	0.02091485629469707	0.6393094665345974	0.5833390658320149	0.9208932395227899	0.03633000858709294	0.03159357255044098	0.1176673567977 9158	0.060069774817634	0.10090277777777777	0.39202220923436587	0.002154353060448612	0.09007773205304069	0.004727866268925536	0.25550850498815764
Candidatus Arthromitus	0	0	0	0	0	0	0	0	0	0	0.019579267519959448	0.00011431184270690443	0	7.177205196296562e-05
Mycoplasma	0	0.07124663930946654	0.005571988718442594	0.0004282655246252676 5	0.0009247638549441839	0.00012081672103419114	0.010121923165401427	0.4860767522993974	6.944444444444444e-05	7.305669199298655e-05	0.5402357115701432	0.0014860539551897576	0.5055439860415377	0.0006459484676666906
Escherichia Shigella	0.008096073404398867	0	0.007910848180504918	0.0022636892015907004	0.02285487812933483	0.00012081672103419114	0.14228203358638142	0.00038058991436726926	0.001875	0.0067212156633547635	6.336332530731213e-05	0.10619570187471422	0.001294534811729611	0.006459484676666906
Enterococcus	0.44467683173660777	0	0.004058609066519915	0.00893239522789844	0.0011889820992139507	0	0.005751092707614446	6.343165239454488e-05	0.000138888888888889	0.07524839275277616	0	0.011545496113397348	5.628412224911353e-05	0.003373286442259384
unclassified Woesearchaeales	0.001012	0.00049	6.87899	0.00036	0.000660	0.351999	0.01265	0	0.14798	0.000876	0.000570	0.001600	0.405808	0.001794

Genus	FKDN3	FKDN1	FKDN2	FKDN4	FKDN5	FKDN6	FKDN7	FKDN8	FKDN9	FKDN10	FKDN11	FKDNs1	FKDNs3	FKDNs4
	0091755498584	52596575633224	8417830364e-05	70847353930866	5456106744171	51673311586	2403956751783		611111111112	6803039158387	2699277658091	365797896662	5214161085	3012990741406
Lysinibacillus	0.009310484415058697	0	0.09025245924193437	0.0007341694707861732	0.4220886452209525	0	0.007821486082355647	0.00012686330478908975	0.09	0.007451782583284629	0	0.00022862368541380886	0.04874204986773231	0.0013636689872963468
unclassified Spirochaetaceae	0.00026986911347996224	0.0003537568982595161	0	0.000611807892321811	0.00019816368320232512	0.44502839192944305	0.00540602714515758	0	0.18756944444444446	0.00021917007597895967	0.0004435432771511849	0.00205761316872428	0	0.002081389506926003
Stenotrophomonas	0.0004722709485899339	0.020517900099051932	0.008873907959001169	0.008626491281737534	0.030385098091023185	0.002597559502235109	0.032781228433402344	0.0032350142721217887	0.051111111111111114	0.12456165984804209	0.0006336332530731213	0.028120713305898493	0.009005459559858164	0.3185243666116414
Delftia	0.10275266495749562	0.0002830055186076129	0.008598748022287954	0.002018966044661976	0.00812471101129533	0.0030204180258547784	0.2533931446974925	0.0012686330478908975	0.001736111111111111	0.07941262419637639	0.0002534533012292485	0.09727937814357568	0.0024202172567118815	0.057920045934113255
Others	0.41249493995412195	0.2073015423800764	0.2871293939602396	0.05432854083817679	0.4772442037122665	0.1653980910958076	0.4121233034276514	0.07992388201712648	0.4186111111111112	0.3128287551139684	0.006082879229501962	0.6596936442615449	0.02240108065514719	0.35161128256656854
Unassigned	0	0.06049	0.00419	0.00079	0	0.000120	0	0.36885	0	0.000584	0.429983	0.001600	0	0.000645

Genus	FKDN3	FKDN1	FKDN2	FKDN4	FKDN5	FKDN6	FKDN7	FKDN8	FKDN9	FKDN10	FKDN11	FKDNs1	FKDNs3	FKDNs4
		242960<br>237724<br>6	6189034<br>876522	5350260<br>0183542		8167210<br>3419114		5058674<br>2785		45353594<br>38924	52553542<br>01	3657978<br>96662		9484676<br>666906

Genus represents each bacterial genus; M24008, M24015, M24016, M24026, etc. represent the relative abundance of each bacterial genus in each sample.

Table S9: Annotation Results of Species at the Species Level

Species	M24008	M24015	M24016	M24026	M24033	M24039
Klebsiella pneumoniae	5.374128943267112e-05	1.838674683288286e-05	0.024552882339624195	4.806459882081518e-05	1.6553550736633007e-05	0.06226542561177
Candidatus Arthromitus sp. SFB rat Yit	0	0.5293360545718646	0.9154952767087201	0.030969623173545246	0.8502400264856812	0.05948806485512686
Escherichia coli	0.9591207925048815	0.08443194145659809	0.0005968429068307641	0.00011215073058190208	0.00038073166694255916	0.4711379672721814
unclassified Mycoplasma	0	0.1771746924816592	0	0.0043258138938733655	0.14211223307399437	0.0018953610568983635
unclassified Woesearchaeales	0	0.00029418794932612575	0	0	0	0
Enterococcus faecium	8.956881572111853e-05	0.0009193373416441429	0.017082055609294284	0.4999038708023584	0.0004800529713623572	0.23080243206725717
Enterococcus faecalis	0	0.0017467409491238716	0	0.2529639835939503	0.00016553550736633007	0.0013511484762047741
Lysinibacillus sphaericus	0	0	0	0	0	0
unclassified Spirochaetaceae	0	0	0	0	0.00016553550736633007	0.00015012760846719713
Stenotrophomonas maltophilia	5.374128943267112e-05	0	4.116157978143201e-05	6.40861317610869e-05	0	0
Others	0.03935653762785	0.206078658502951	0.041737841898372	0.211612407075109	0.0064393312365502	0.17290947305

Species	M24008	M24015	M24016	M24026	M24033	M24039
	946	04	03		39	209442
Unassigned	0.0013256184726725543	0	0.0004939389573771841	0	0	0

Species	FKDN3	FKDN1	FKDN2	FKDN4	FKDN5	FKDN6	FKDN7	FKDN8	FKDN9	FKDN10	FKDN11	FKDNs1	FKDNs3	FKDNs4
Klebsiella pneumoniae	0.02091485629469707	0.6393094665345974	0.5833390658320149	0.9208932395227899	0.03633000858709294	0.03159357255044098	0.11766735679779158	0.060069774817634	0.10090277777777777	0.39202220923436587	0.002154353060448612	0.09007773205304069	0.004727866268925536	0.25550850498815764
Candidatus_ Arthromitus sp. SFB rat Yit	0	0	0	0	0	0	0	0	0	0	0.019579267519959448	0.00011431184270690443	0	7.177205196296562e-05
Escherichia coli	0.008096073404398867	0	0.007910848180504918	0.0022636892015907004	0.02285487812933483	0.00012081672103419114	0.14228203358638142	0.00038058991436726926	0.001875	0.0067212156633547635	6.336332530731213e-05	0.10619570187471422	0.001294534811729611	0.006459484676666906
unclassified Mycoplasma	0	0.07068062827225131	0.005159248813372773	0.00042826552462526765	0	0.00012081672103419114	0	0.48449096098953376	0	0	0.5402357115701432	0.0010288065843621 4	0	0.0006459484676666906
unclassified Woesearchaeales	0.0010120091755498584	0.0004952596575633224	6.878998417830364e-05	0.0003670847353930866	0.0006605456106744171	0.35199951673311586	0.012652403956751783	0	0.14798611111111112	0.0008766803039158387	0.0005702699277658091	0.001600365797896662	0.4058085214161085	0.0017943012990741406

Species	FKDN3	FKDN1	FKDN2	FKDN4	FKDN5	FKDN6	FKDN7	FKDN8	FKDN9	FKDN10	FKDN11	FKDNs1	FKDNs3	FKDNs4
Enterococcus faecium	0.00020240183510997166	0	0.00020636995253491092	6.11807892321811e-05	0.0005944910496069754	0	0.004945939728548425	0	0.000138888888888889	0.04514903565166569	0	0.011431184270690443	0	0
Enterococcus faecalis	0.44447442990149777	0	0.0038522391139850037	0.008871214438666258	0.0005944910496069754	0	0.0008051529790660225	6.343165239454488e-05	0	0.03009935710111046	0	0.00011431184270690443	5.628412224911353e-05	0.003373286442259384
Lysinibacillus sphaericus	0.009310484415058697	0	0.09018366925775607	0.0007341694707861732	0.4219565360988176	0	0.007821486082355647	0.00012686330478908975	0.08743055555555555	0.0037258912916423143	0	0.00011431184270690443	0.04874204986773231	0.0013636689872963468
unclassified Spirochaetaceae	0.00026986911347996224	0.0003537568982595161	0	0.000611807892321811	0.00019816368320232512	0.44502839192944305	0.00540602714515758	0	0.18756944444444446	0.00021917007597895967	0.0004435432771511849	0.00205761316872428	0	0.002081389506926003
Stenotrophomonas maltophilia	0.0004722709485899339	0.020517900099051932	0.008873907959001169	0.008626491281737534	0.030385098091023185	0.002597559502235109	0.032781228433402344	0.0032350142721217887	0.051111111111111114	0.12456165984804209	0.0006336332530731213	0.028120713305898493	0.009005459559858164	0.3185243666116414
Others	0.5152476049116184	0.20815055893589912	0.2962096718717759	0.056347506882838766	0.4864257877006411	0.16841850912166245	0.6756383712905443	0.082778306374881	0.42298611111111123	0.3960403272939801	0.006336332530731208	0.7575445816186561	0.5303652839533971	0.4095313285006816
Unassigned	0	0.06049	0.00419	0.00079	0	0.000120	0	0.36885	0	0.000584	0.429983	0.001600	0	0.000645

Species	FKDN3	FKDN1	FKDN2	FKDN4	FKDN5	FKDN6	FKDN7	FKDN8	FKDN9	FKDN10	FKDN11	FKDNs1	FKDNs3	FKDNs4
		2429602<br>377246	6189034<br>876522	5350260<br>0183542		8167210<br>3419114		505867<br>42785		45353594<br>38924	52553542<br>01	3657978<br>96662		9484676<br>666906

Species represents each bacterial species; M24008, M24015, M24016, M24026, etc. represent the relative abundance of each bacterial species in each sample.

Table S10. Lefse Statistical Results Table

Biomarker names	Logarithm value	Groups	LDA scores	*P* values
k Bacteria.p Firmicutes.c Bacilli.o Erysipelotrichales.f Erysipelotrichaceae.g Turicibacter	4.410809442	FKDN	4.26572125	0.0267223 628609
k Bacteria.p Proteobacteria.c Gammaproteobacteria.o Xanthomonadales.f Xanthomonadaceae.g Stenotrophomonas	4.664869071	FKDN	4.276426571	0.0005217 72580871
k Bacteria.p Proteobacteria.c Gammaproteobacteria.o Enterobacterales.f Pseudoalteromonadaceae.g Pseudoalteromonas.s Ruegeria sp	4.453163933	FKDN	4.156193987	0.0421185 282205
k Bacteria.p Proteobacteria.c Gammaproteobacteria.o Enterobacterales.f Morganellaceae	4.357875975	FKDN	4.006074588	0.0011905 7931186
k Bacteria.p Firmicutes.c Bacilli.o Bacillales.f Planococcaceae.g Lysinibacillus.s Lysinibacillus sphaericus	4.683423877	FKDN	4.407967944	0.0043231 1122553
k Bacteria.p Firmicutes.c Bacilli.o Bacillales.f Planococcaceae	4.693210207	FKDN	4.416116122	0.0058097 5308973
k Bacteria.p Proteobacteria.c Gammaproteobacteria.o Burkholderiales.f Comamonadaceae.g Delftia.s Delftia acidovorans	4.649230167	FKDN	4.301573611	0.0004470 93742368
k Bacteria.p Proteobacteria.c Gammaproteobacteria.o Xanthomonadales.f Xanthomonadaceae.g Stenotrophomonas.s Stenotrophomonas maltophilia	4.664869071	FKDN	4.276436376	0.0005217 72580871
k Archaea.p unclassified Archaea.c unclassified Archaea.o unclassified Archaea	4.315431127	FKDN	4.009164459	0.0030043 3238804
k Bacteria.p Proteobacteria.c Gammaproteobacteria.o Enterobacterales.f Pseudoalteromonadaceae	4.453163933	FKDN	4.156192891	0.0421185 282205
k Archaea.p Nanoarchaeota.c Nanoarchaeia.o Woesearchaeales	4.82309008	FKDN	4.5819942	0.0017216

Biomarker names	Logarithm value	Groups	LDA scores	*P* values
				7317401
k Bacteria.p Spirochaetota.c Spirochaetia.o Spirochaetales.f Spirochaetaceae.g unclassified Spirochaetaceae.s unclassified Spirochaetaceae	4.663811968	FKDN	4.43257097	0.0114663 723265
k Bacteria.p Spirochaetota.c Spirochaetia.o Spirochaetales.f Spirochaetaceae.g unclassified Spirochaetaceae	4.663811968	FKDN	4.432575666	0.0114663 723265
k Bacteria.p Proteobacteria.c Gammaproteobacteria.o Enterobacterales.f Enterobacteriaceae.g Klebsiella.s Klebsiella pneumoniae	5.380394995	FKDN	5.040317783	0.0050430 2260381
k Archaea.p unclassified Archaea.c unclassified Archaea.o unclassified Archaea.f unclassified Archaea.g unclassified Archaea.s unclassified Archaea	4.315431127	FKDN	4.009249436	0.0030043 3238804
k Archaea.p Nanoarchaeota.c Nanoarchaeia.o Woesearchaeales.f unclassified Woesearchaeales	4.821358459	FKDN	4.580752453	0.0017216 7317401
k Bacteria.p Proteobacteria.c Gammaproteobacteria.o Enterobacterales.f Enterobacteriaceae.g Klebsiella	5.380394995	FKDN	5.040317783	0.0050430 2260381
k Bacteria.p Proteobacteria.c Gammaproteobacteria.o Burkholderiales.f Comamonadaceae	4.806958384	FKDN	4.449495957	0.0207291 434952
k Archaea.p unclassified Archaea.c unclassified Archaea	4.315431127	FKDN	4.009241319	0.0030043 3238804
k Bacteria.p Firmicutes.c Bacilli.o Erysipelotrichales.f Erysipelotrichaceae	4.410809442	FKDN	4.266509444	0.0267223 628609
k Bacteria.p Spirochaetota.c Spirochaetia.o Spirochaetales	4.663811968	FKDN	4.432557293	0.0114663 723265
k Bacteria.p Proteobacteria.c Gammaproteobacteria.o Burkholderiales.f Comamonadaceae.g Delftia	4.649230167	FKDN	4.301573657	0.0004470

Biomarker names	Logarithm value	Groups	LDA scores	*P* values
				93742368
k Bacteria.p Spirochaetota.c Spirochaetia.o Spirochaetales.f Spirochaetaceae	4.663811968	FKDN	4.432578619	0.0114663 723265
k Bacteria.p Proteobacteria.c Gammaproteobacteria.o Xanthomonadales	4.678288217	FKDN	4.289757205	0.0005320 05505139
k Bacteria.p Firmicutes.c Bacilli.o Bacillales.f Planococcaceae.g Lysinibacillus	4.687680982	FKDN	4.411028905	0.0043231 1122553
k Archaea.p Nanoarchaeota	4.82309008	FKDN	4.581993169	0.0017216 7317401
k Bacteria.p Proteobacteria.c Gammaproteobacteria.o Xanthomonadales.f Xanthomonadaceae	4.678288217	FKDN	4.289757205	0.0005320 05505139
k Bacteria.p Spirochaetota	4.663811968	FKDN	4.432567574	0.0114663 723265
k Bacteria.p Firmicutes.c Bacilli.o Erysipelotrichales	4.410809442	FKDN	4.183270461	0.0170338 391393
k Archaea.p unclassified Archaea.c unclassified Archaea.o unclassified Archaea.f unclassified Archaea.g unclassified Archaea	4.315431127	FKDN	4.008982828	0.0030043 3238804
k Bacteria.p Firmicutes.c Bacilli.o Erysipelotrichales.f Erysipelotrichaceae.g Turicibacter.s Turicibacter sanguinis	4.410809442	FKDN	4.27092581	0.0267223 628609
k Archaea.p unclassified Archaea	4.315431127	FKDN	4.009125621	0.0030043 3238804
k Bacteria.p Proteobacteria.c Gammaproteobacteria.o Enterobacterales.f Pseudoalteromonadaceae.g	4.453163933	FKDN	4.156195811	0.0421185

Biomarker names	Logarithm value	Groups	LDA scores	*P* values
Pseudoalteromonas				282205
k Bacteria.p Spirochaetota.c Spirochaetia	4.663811968	FKDN	4.43256116	0.0114663 723265
k Archaea.p Nanoarchaeota.c Nanoarchaeia.o Woesearchaeales.f unclassified Woesearchaeales.g unclassified Woesearchaeales.s unclassified Woesearchaeales	4.821358459	FKDN	4.580739395	0.0017216 7317401
k Bacteria.p Proteobacteria.c Gammaproteobacteria.o Burkholderiales	4.8722517	FKDN	4.524232622	0.0133475 759268
k Bacteria.p Firmicutes.c Bacilli.o Bacillales	4.77394967	FKDN	4.472991976	0.0167623 361772
k Archaea.p Nanoarchaeota.c Nanoarchaeia	4.82309008	FKDN	4.58195688	0.0017216 7317401
k Archaea.p Nanoarchaeota.c Nanoarchaeia.o Woesearchaeales.f unclassified Woesearchaeales.g unclassified Woesearchaeales	4.821358459	FKDN	4.580771289	0.0017216 7317401
k Archaea	4.951393447	FKDN	4.692497347	0.0006559 9562893
k Archaea.p unclassified Archaea.c unclassified Archaea.o unclassified Archaea.f unclassified Archaea	4.315431127	FKDN	4.009151792	0.0030043 3238804
k Bacteria.p Firmicutes.c Clostridia.o Clostridiales.f Clostridiaceae.g Candidatus Arthromitus	5.60356515	M	5.249069136	0.0024820 4635789
k Bacteria.p Firmicutes.c Clostridia.o Clostridiales.f Clostridiaceae	5.615458906	M	5.25819728	0.0047192 4036688
k Bacteria	5.999946186	M	4.817802221	0.0006559

Biomarker names	Logarithm value	Groups	LDA scores	*P* values
				9562893
k Bacteria.p Firmicutes.c Bacilli.o Lactobacillales.f Enterococcaceae.g Enterococcus.s Enterococcus faecium	5.097716365	M	4.813698159	0.0240196132288
k Bacteria.p Firmicutes.c Clostridia.o Clostridiales	5.615458906	M	5.25819728	0.00471924036688
k Bacteria.p Firmicutes.c Clostridia	5.623624836	M	5.259066955	0.0105337059466
k Bacteria.p Firmicutes.c Clostridia.o Clostridiales.f Clostridiaceae.g Candidatus Arthromitus.s Candidatus Arthromitus sp SFB rat Yit	5.60356515	M	5.249006754	0.00248204635789

The first column represents the species name, and the following four columns represent the mean relative abundance within each taxonomic unit at the highest level (these are the logarithmic transformed values, the second column). The corresponding group names (the third column), the LDA effect size (default requirement greater than 4), and the P value (default less than 0.05) are also provided. If the species does not show significant differences between groups, the latter three columns will be empty. Generally, based on the LDA effect size in the fourth column and the P value in the fifth column, the species that represent significant differences between groups can be identified.

Table S11: Metastats Statistical Results of Species Levels

species	Mean (FKDN)	Variance (FKDN)	Std.err (FKDN)	Mean (M)	Variance (M)	Std.err (M)	P value	Q value
Lactobacillus johnsonii	1.04e-05	1.52e-09	1.04e-05	2.27e-04	2.07e-08	5.87e-05	0.00e+00	0.00e+00
unclassified unidentified rumen bacterium RF39	0.00e+00	0.00e+00	0.00e+00	1.06e-04	4.56e-09	2.76e-05	0.00e+00	0.00e+00
Devosia psychrophila	7.98e-05	3.64e-08	5.10e-05	0.00e+00	0.00e+00	0.00e+00	2.83e-06	1.35e-04
unclassified Gemmatimonas	6.66e-05	6.22e-08	6.66e-05	0.00e+00	0.00e+00	0.00e+00	2.83e-06	1.35e-04
unclassified Pedobacter	9.32e-05	4.98e-08	5.96e-05	0.00e+00	0.00e+00	0.00e+00	2.83e-06	1.35e-04
Burkholderia cepacia	4.72e-06	3.12e-10	4.72e-06	2.73e-05	1.21e-10	4.49e-06	3.56e-06	1.35e-04
Lactiplantibacillus plantarum	2.83e-03	8.79e-06	7.93e-04	1.11e-04	5.98e-08	9.98e-05	3.56e-06	1.35e-04
unclassified Akkermansia	0.00e+00	0.00e+00	0.00e+00	6.17e-05	2.15e-09	1.89e-05	3.56e-06	1.35e-04
uncultured rumen bacterium	6.39e-05	5.71e-08	6.39e-05	3.74e-04	2.62e-08	6.61e-05	3.56e-06	1.35e-04
Microbacterium hatanonis	5.96e-05	1.15e-08	2.86e-05	0.00e+00	0.00e+00	0.00e+00	7.55e-06	2.57e-04
uncultured rumen bacterium 3C0d 6	0.00e+00	0.00e+00	0.00e+00	2.38e-04	3.35e-08	7.47e-05	1.42e-05	4.41e-04
Photobacterium kishitanii	0.00e+00	0.00e+00	0.00e+00	1.06e-04	6.75e-09	3.35e-05	1.78e-05	4.91e-04
Arthrobacter sp. LM3	8.67e-05	9.28e-08	8.14e-05	0.00e+00	0.00e+00	0.00e+00	2.02e-05	4.91e-04
uncultured Aciditerrimonas sp.	5.64e-05	4.45e-08	5.64e-05	0.00e+00	0.00e+00	0.00e+00	2.02e-05	4.91e-04
Carnobacterium divergens	1.28e-03	2.27e-06	4.03e-04	3.34e-05	2.93e-09	2.21e-05	4.27e-05	9.71e-04
Fluviicola bacterium SH1 3	8.17e-05	7.52e-08	7.33e-05	0.00e+00	0.00e+00	0.00e+00	5.39e-05	1.02e-03
Paeniglutamicibacter sulfureus	8.17e-05	9.33e-08	8.17e-05	0.00e+00	0.00e+00	0.00e+00	5.39e-05	1.02e-03
unclassified TRA3 20	4.72e-05	3.12e-08	4.72e-05	0.00e+00	0.00e+00	0.00e+00	5.39e-05	1.02e-03

species	Mean (FKDN)	Variance (FKDN)	Std.err (FKDN)	Mean (M)	Variance (M)	Std.err (M)	P value	Q value
unclassified Clostridium sensu stricto 1	1.22e-05	1.09e-09	8.82e-06	1.20e-04	7.14e-09	3.45e-05	9.61e-05	1.72e-03
Alkanindiges hydrocarboniclasticus	6.35e-05	3.37e-08	4.90e-05	0.00e+00	0.00e+00	0.00e+00	1.44e-04	1.96e-03
Anaerolineae bacterium UTCFX1	7.35e-05	7.56e-08	7.35e-05	0.00e+00	0.00e+00	0.00e+00	1.44e-04	1.96e-03
Aquirufa nivalisilvae	7.35e-05	7.56e-08	7.35e-05	0.00e+00	0.00e+00	0.00e+00	1.44e-04	1.96e-03
Cupriavidus metallidurans	4.86e-05	2.31e-08	4.06e-05	0.00e+00	0.00e+00	0.00e+00	1.44e-04	1.96e-03
Leptotrichia sp. oral taxon 215 str. W9775	4.41e-05	2.15e-08	3.92e-05	0.00e+00	0.00e+00	0.00e+00	1.44e-04	1.96e-03
unclassified Sphingomonas	4.25e-05	2.52e-08	4.25e-05	0.00e+00	0.00e+00	0.00e+00	1.44e-04	1.96e-03
Lactobacillus reuteri	0.00e+00	0.00e+00	0.00e+00	2.11e-04	3.01e-08	7.08e-05	3.27e-04	4.29e-03
Ellin6055 bacterium Ellin6055	3.47e-05	8.63e-09	2.48e-05	0.00e+00	0.00e+00	0.00e+00	3.85e-04	4.53e-03
Megasphaera micronuciformis	4.22e-05	1.25e-08	2.99e-05	0.00e+00	0.00e+00	0.00e+00	3.85e-04	4.53e-03
unclassified Candidatus Fonsibacter ubiquis	5.53e-05	2.50e-08	4.22e-05	0.00e+00	0.00e+00	0.00e+00	3.85e-04	4.53e-03
unclassified RF39	0.00e+00	0.00e+00	0.00e+00	1.70e-04	2.13e-08	5.96e-05	6.65e-04	7.56e-03
Klebsiella pneumoniae	2.33e-01	8.44e-02	7.77e-02	1.45e-02	6.44e-04	1.04e-02	7.97e-04	8.49e-03
unclassified NK4A214 group	0.00e+00	0.00e+00	0.00e+00	5.47e-05	2.34e-09	1.97e-05	7.97e-04	8.49e-03
unclassified UCG 004	0.00e+00	0.00e+00	0.00e+00	2.41e-05	4.82e-10	8.97e-06	8.61e-04	8.56e-03
rumen bacterium NK4A214	0.00e+00	0.00e+00	0.00e+00	8.15e-05	5.50e-09	3.03e-05	8.65e-04	8.56e-03
unclassified p 2534 18B5 gut group	0.00e+00	0.00e+00	0.00e+00	1.31e-04	1.42e-08	4.87e-05	8.97e-04	8.56e-03
Providencia alcalifaciens	1.84e-02	6.28e-04	6.70e-03	4.13e-04	1.02e-06	4.13e-04	9.04e-04	8.56e-03
Aquabacterium olei	3.32e-05	7.50e-09	2.32e-05	0.00e+00	0.00e+00	0.00e+00	1.03e-03	8.99e-03

species	Mean (FKDN)	Variance (FKDN)	Std.err (FKDN)	Mean (M)	Variance (M)	Std.err (M)	P value	Q value
Moraxella catarrhalis	5.75e-05	3.41e-08	4.93e-05	0.00e+00	0.00e+00	0.00e+00	1.03e-03	8.99e-03
Sphingomonas astaxanthinifaciens	5.72e-05	4.57e-08	5.72e-05	0.00e+00	0.00e+00	0.00e+00	1.03e-03	8.99e-03
Sedimenticola selenatireducens	0.00e+00	0.00e+00	0.00e+00	3.07e-05	9.15e-10	1.24e-05	1.31e-03	1.12e-02
Ruminococcus sp.	0.00e+00	0.00e+00	0.00e+00	3.27e-05	1.07e-09	1.34e-05	1.68e-03	1.39e-02
Meiothermus silvanus	3.73e-03	3.37e-05	1.55e-03	0.00e+00	0.00e+00	0.00e+00	1.80e-03	1.46e-02
unclassified Christensenellaceae R 7 group	0.00e+00	0.00e+00	0.00e+00	1.44e-04	2.17e-08	6.02e-05	1.87e-03	1.48e-02
Brevundimonas vesicularis	7.46e-04	1.39e-06	3.15e-04	9.48e-06	1.09e-10	4.26e-06	2.18e-03	1.69e-02
Delftia acidovorans	4.42e-02	5.13e-03	1.91e-02	0.00e+00	0.00e+00	0.00e+00	2.68e-03	1.82e-02
Cellvibrio gandavensis	4.90e-05	3.36e-08	4.90e-05	0.00e+00	0.00e+00	0.00e+00	2.75e-03	1.82e-02
Lawsonella clevelandensis	2.71e-05	5.16e-09	1.92e-05	0.00e+00	0.00e+00	0.00e+00	2.75e-03	1.82e-02
Limnobacter thiooxidans	3.03e-05	1.29e-08	3.03e-05	0.00e+00	0.00e+00	0.00e+00	2.75e-03	1.82e-02
Methyloferula sp. PAMC 26571	2.41e-05	8.15e-09	2.41e-05	0.00e+00	0.00e+00	0.00e+00	2.75e-03	1.82e-02
Psychrobacter phenylpyruvicus	2.66e-05	6.77e-09	2.20e-05	0.00e+00	0.00e+00	0.00e+00	2.75e-03	1.82e-02
Sphingobium yanoikuyae	2.91e-05	4.15e-09	1.72e-05	0.00e+00	0.00e+00	0.00e+00	2.75e-03	1.82e-02
Candidatus Arthromitus sp. SFB rat Yit	1.41e-03	2.73e-05	1.40e-03	3.98e-01	1.79e-01	1.73e-01	2.78e-03	1.82e-02
uncultured Firmicutes bacterium	0.00e+00	0.00e+00	0.00e+00	2.84e-05	9.78e-10	1.28e-05	4.13e-03	2.66e-02
Dechloromonas agitata	2.47e-05	5.81e-09	2.04e-05	0.00e+00	0.00e+00	0.00e+00	7.34e-03	4.39e-02
Geminicoccus roseus	2.27e-05	7.18e-09	2.27e-05	0.00e+00	0.00e+00	0.00e+00	7.34e-03	4.39e-02
Immundisolibacter cernigliae	2.48e-05	8.61e-09	2.48e-05	0.00e+00	0.00e+00	0.00e+00	7.34e-03	4.39e-02

species	Mean (FKDN)	Variance (FKDN)	Std.err (FKDN)	Mean (M)	Variance (M)	Std.err (M)	P value	Q value
unclassified Sphingomonadaceae	4.08e-05	2.33e-08	4.08e-05	0.00e+00	0.00e+00	0.00e+00	7.34e-03	4.39e-02
unclassified Archaea	2.05e-02	1.33e-03	9.74e-03	7.35e-05	3.25e-08	7.35e-05	8.08e-03	4.75e-02
Stenotrophomonas maltophilia	4.57e-02	7.22e-03	2.27e-02	2.65e-05	8.95e-10	1.22e-05	1.12e-02	6.48e-02
Pseudomonas azotoformans	3.71e-03	4.78e-05	1.85e-03	2.89e-05	1.67e-09	1.67e-05	1.21e-02	6.90e-02
Methylorubrum extorquens	4.79e-05	1.77e-08	3.55e-05	1.03e-05	6.35e-10	1.03e-05	1.29e-02	7.22e-02
unclassified UCG 002	4.72e-06	3.12e-10	4.72e-06	1.17e-04	2.03e-08	5.81e-05	1.53e-02	8.41e-02
Lactobacillus sakei	1.19e-04	5.40e-08	6.21e-05	0.00e+00	0.00e+00	0.00e+00	1.55e-02	8.41e-02
Pseudomonas stutzeri	3.87e-04	5.81e-07	2.04e-04	0.00e+00	0.00e+00	0.00e+00	1.62e-02	8.66e-02
Cenchrus americanus	1.91e-02	9.61e-04	8.28e-03	3.09e-03	2.41e-05	2.00e-03	1.84e-02	9.56e-02
Leuconostoc citreum	3.81e-05	4.98e-09	1.89e-05	4.80e-04	3.30e-07	2.34e-04	1.85e-02	9.56e-02
Arthrobacter parietis	3.27e-05	1.49e-08	3.27e-05	0.00e+00	0.00e+00	0.00e+00	1.96e-02	9.56e-02
Flavobacteria bacterium MS024 3C	3.27e-05	1.49e-08	3.27e-05	0.00e+00	0.00e+00	0.00e+00	1.96e-02	9.56e-02
Knoellia locipacati	3.27e-05	1.49e-08	3.27e-05	0.00e+00	0.00e+00	0.00e+00	1.96e-02	9.56e-02
Sphingomonas sp. CC MHH0546	1.98e-05	5.51e-09	1.98e-05	0.00e+00	0.00e+00	0.00e+00	1.96e-02	9.56e-02
Corynebacterium pilbarense	4.78e-04	9.54e-07	2.61e-04	0.00e+00	0.00e+00	0.00e+00	2.25e-02	1.08e-01
Achromobacter xylosoxidans	2.74e-03	3.03e-05	1.47e-03	6.34e-05	4.45e-09	2.72e-05	2.38e-02	1.13e-01
Veillonella dispar	3.76e-04	6.11e-07	2.09e-04	0.00e+00	0.00e+00	0.00e+00	2.56e-02	1.20e-01
Pseudomonas argentinensis	6.82e-04	1.98e-06	3.76e-04	9.48e-06	1.09e-10	4.26e-06	2.64e-02	1.20e-01
unclassified Woesearchaeales	6.61e-02	1.92e-02	3.70e-02	4.90e-05	1.44e-08	4.90e-05	2.64e-02	1.20e-01

species	Mean (FKDN)	Variance (FKDN)	Std.err (FKDN)	Mean (M)	Variance (M)	Std.err (M)	P value	Q value
Sphingopyxis chilensis	1.15e-04	5.86e-08	6.47e-05	0.00e+00	0.00e+00	0.00e+00	2.69e-02	1.21e-01
Ruminococcaceae bacterium P7	0.00e+00	0.00e+00	0.00e+00	6.42e-05	7.96e-09	3.64e-05	2.83e-02	1.25e-01
unclassified Odoribacter	0.00e+00	0.00e+00	0.00e+00	4.12e-05	3.30e-09	2.34e-05	2.86e-02	1.25e-01
Alcaligenes faecalis	4.40e-04	8.96e-07	2.53e-04	0.00e+00	0.00e+00	0.00e+00	2.97e-02	1.28e-01
Rubellimicrobium roseum	4.93e-04	1.17e-06	2.89e-04	0.00e+00	0.00e+00	0.00e+00	3.30e-02	1.41e-01
beta proteobacterium R 43960	9.34e-05	3.95e-08	5.31e-05	2.99e-06	5.35e-11	2.99e-06	3.56e-02	1.50e-01
Staphylococcus hominis	4.19e-03	8.67e-05	2.49e-03	0.00e+00	0.00e+00	0.00e+00	3.71e-02	1.54e-01
Sphingopyxis solisilvae	1.15e-04	6.58e-08	6.86e-05	0.00e+00	0.00e+00	0.00e+00	3.74e-02	1.54e-01
Lactococcus lactis	3.82e-03	7.34e-05	2.29e-03	9.38e-06	5.28e-10	9.38e-06	3.94e-02	1.60e-01
Ruminococcus flavefaciens	0.00e+00	0.00e+00	0.00e+00	1.48e-04	4.80e-08	8.95e-05	3.99e-02	1.60e-01
Bifidobacterium pseudocatenulatum	0.00e+00	0.00e+00	0.00e+00	1.01e-04	2.32e-08	6.22e-05	4.24e-02	1.68e-01
Propionibacteriaceae bacterium	3.40e-03	5.51e-05	1.98e-03	1.90e-04	1.56e-07	1.61e-04	4.30e-02	1.68e-01
unclassified Ruminococcaceae	0.00e+00	0.00e+00	0.00e+00	1.91e-04	8.50e-08	1.19e-04	4.42e-02	1.68e-01
Shimia aestuarii	0.00e+00	0.00e+00	0.00e+00	1.39e-04	4.51e-08	8.67e-05	4.43e-02	1.68e-01
Lysinibacillus sphaericus	4.80e-02	1.26e-02	3.00e-02	0.00e+00	0.00e+00	0.00e+00	4.62e-02	1.68e-01
Bacillus cereus	4.75e-04	1.25e-06	2.98e-04	0.00e+00	0.00e+00	0.00e+00	4.85e-02	1.68e-01
Alteromonas macleodii	1.45e-04	1.17e-07	9.15e-05	0.00e+00	0.00e+00	0.00e+00	4.89e-02	1.68e-01
Marinobacter nauticus	0.00e+00	0.00e+00	0.00e+00	5.43e-05	7.08e-09	3.44e-05	4.98e-02	1.68e-01

The first column contains the species classification information; the second to fourth columns respectively represent the average values, variances and standard

deviations of the first group; the fifth to seventh columns respectively represent the average values, variances and standard deviations of the second group; P value is the p-value of the hypothesis test, and it is generally considered that a value less than 0.05 indicates a significant difference; Q value is the corrected q-value of the P value.

Table S12. KEGG Function Annotation Results

Class1 name	Class2 name	Class3 name	M24 008	M24 015	M24 016	M24 026	M24 033	M24 039	FK DN 3	FK DN 1	FK DN 2	FK DN 4	FK DN 5	FK DN 6	FK DN 7	FK DN 8	FK DN 9	FK DN 10	FK DN 11	FK DN s1	FK DN s3	FK DN s4
Metabolism	Carbohydrate metabolism	Galactose metabolism	671126	284579	136546	857003	183801	662245	160412	150516	147333	221679	71278	47589	44288	94926	61287	117855	86821	52306	4819	98010
Metabolism	Lipid metabolism	Fatty acid elongation	0	0	1	0	0	0	0	0	0	0	0	0	0	1	0	0	0	7	0	505
Metabolism	Lipid metabolism	Steroid biosynthesis	9	827	381	5	7	9	386	21	1496	11	3871	3	401	22	3178	234	1	212	514	21
Metabolism	Lipid metabolism	Primary bile acid biosynthesis	18	175	12	1	9	3	419	74	32	15	76	20	623	32	52	385	3	370	14	334
Metabolism	Lipid metabolism	Secondary bile acid biosynthesis	2946	7783	940	36668	209	19921	5245	206	1034	462	7582	124	910	598	1248	1984	53	593	797	3595
Metabolism	Energy metabolism	Photosynthesis - antenna proteins	0	7356	4332	0	0	12	2381	180	9372	0	2976	0	4294	252	29100	1656	12	2365	12	0
Metabolism	Xenobiotics biodegradation and metabolism	Fluorobenzoate degradation	35992	5686	10385	65	159	43425	3677	46422	39415	68953	3878	2410	8691	4618	10538	27503	219	9628	509	20906
Metabolism	Biosynthesis of other secondary	Staurosporine biosynthesis	66	241	71	2	19	31	1	99	108	27	3	5	1203	123	50	99	0	2988	5	253

Class1 name	Class2 name	Class3 name	M24008	M24015	M24016	M24026	M24033	M24039	FKDN3	FKDN1	FKDN2	FKDN4	FKDN5	FKDN6	FKDN7	FKDN8	FKDN9	FKDN10	FKDN11	FKDNs1	FKDNs3	FKDNs4
	metabolites																					
Metabolism	Metabolism of other amino acids	D-Arginine and D-ornithine metabolism	131	322	154	18	67	512	481	536	916	106	4067	36	755	28	1028	977	11	1739	548	596
Metabolism	Glycan biosynthesis and metabolism	N-Glycan biosynthesis	338	572	447	35	88	88	1030	820	735	240	617	8111	562	134	3976	2413	32	901	185	6512
Metabolism	Glycan biosynthesis and metabolism	Other glycan degradation	57148	22440	25415	90076	21043	96695	20308	16490	17715	26774	6330	1070	5148	1895	6274	15342	285	7650	475	17524
Metabolism	Glycan biosynthesis and metabolism	Other types of O-glycan biosynthesis	0	0	0	0	0	0	0	0	23	0	0	0	0	0	0	0	0	0	0	0
Metabolism	Glycan biosynthesis and metabolism	Mannose type O-glycan biosynthesis	0	0	0	0	0	0	0	0	23	0	0	0	0	0	0	0	0	0	0	0

Class1 name	Class2 name	Class3 name	M24008	M24015	M24016	M24026	M24033	M24039	FKDN3	FKDN1	FKDN2	FKDN4	FKDN5	FKDN6	FKDN7	FKDN8	FKDN9	FKDN10	FKDN11	FKDNs1	FKDNs3	FKDNs4
Metabolism	Glycan biosynthesis and metabolism	Glycosaminoglycan degradation	38849	11279	12279	37129	10611	67573	7239	6663	6581	9295	1356	7827	3946	1316	5237	7513	133	6664	437	10289
Metabolism	Xenobiotics biodegradation and metabolism	Polycyclic aromatic hydrocarbon degradation	123	670	2782	28	87	6674	2871	12123	9800	17249	985	680	4268	1192	2226	7918	68	3348	159	5937
Metabolism	Xenobiotics biodegradation and metabolism	Atrazine degradation	707	3543	5372	60	196	11284	3528	18870	16555	25874	1731	2327	5872	1805	8034	11286	102	7123	213	7666
Metabolism	Metabolism of terpenoids and polyketides	Sesquiterpenoid and triterpenoid biosynthesis	35	1482	764	9	12	16	584	47	2273	12	4142	0	843	65	5645	430	2	386	521	69
Metabolism	Biosynthesis of other secondary metabolites	Flavonoid biosynthesis	2	5759	8901	388	10274	635	1	3	39	7	341	25	3	0	0	74	40	31	0	0

Class1 name	Class2 name	Class3 name	M24008	M24015	M24016	M24026	M24033	M24039	FKDN3	FKDN1	FKDN2	FKDN4	FKDN5	FKDN6	FKDN7	FKDN8	FKDN9	FKDN10	FKDN11	FKDNs1	FKDNs3	FKDNs4
Metabolism	Biosynthesis of other secondary metabolites	Stilbenoid, diarylheptanoid and gingerol biosynthesis	56	5933	9047	389	10278	703	1	102	42	9	371	49	68	6	8	263	41	273	1	29
Metabolism	Biosynthesis of other secondary metabolites	Betalain biosynthesis	30	22	11	0	4	2	4	57	13	7	0	10	109	3	27	86	5	213	0	65
Metabolism	Xenobiotics biodegradation and metabolism	Steroid degradation	0	181	0	0	0	0	78	31	701	10	3616	17	40	6	715	98	0	63	506	535
Metabolism	Metabolism of terpenoids and polyketides	Biosynthesis of type II polyketide products	1	3	12	1	16	1	0	4	0	2	1	0	3	0	0	57	0	33	0	0
Human Diseases	Drug resistance: Antineoplastic	Platinum drug resistance	54510	10567	9377	13206	211	42666	6538	37165	28636	45105	7205	2201	13867	6139	21003	30006	302	16046	1993	50498

Class1 name	Class2 name	Class3 name	M24008	M24015	M24016	M24026	M24033	M24039	FKDN3	FKDN1	FKDN2	FKDN4	FKDN5	FKDN6	FKDN7	FKDN8	FKDN9	FKDN10	FKDN11	FKDNs1	FKDNs3	FKDNs4
Cellular Processes	Cell motility	Flagellar assembly	687305	268522	286429	404987	312966	353899	23247	62308	39921	14631	141853	201366	67092	26202	125614	82994	2254	104136	26342	152643
Genetic Information Processing	Transcription	Basal transcription factors	30	313	117	29	26	2956	856	31	317	53	73	7382	386	8	2744	431	13	353	6	381
Genetic Information Processing	Replication and repair	Non-homologous end-joining	103	1035	423	45	64	107	923	2453	2609	454	8310	129	1298	262	3270	5250	40	1797	1375	9966
Environmental Information Processing	Signal transduction	Ras signaling pathway	0	2	0	0	0	0	0	3	0	0	0	0	0	0	0	0	0	2	0	0
Environmental Information Processing	Signal transduction	Rap1 signaling pathway	0	0	0	0	0	0	0	0	0	0	0	0	0	0	0	0	0	2	0	0
Environmental Information Processing	Signal transduction	Calcium signaling pathway	0	7	1	0	0	0	0	0	0	0	0	0	2	0	26	0	0	42	0	0

Class1 name	Class2 name	Class3 name	M24 008	M24 015	M24 016	M24 026	M24 033	M24 039	FKDN 3	FKDN 1	FKDN 2	FKDN 4	FKDN 5	FKDN 6	FKDN 7	FKDN 8	FKDN 9	FKDN 10	FKDN 11	FKDN s1	FKDN s3	FKDN s4
Environmental Information Processing	Signal transduction	cGMP-PKG signaling pathway	0	0	0	0	0	0	0	0	0	0	0	0	0	0	0	0	0	2	0	0
Environmental Information Processing	Signal transduction	cAMP signaling pathway	5	142	81	3	34	12	48	19	306	0	0	7	3	0	0	24	0	154	0	22
Environmental Information Processing	Signal transduction	Plant hormone signal transduction	3	0	108	0	0	0	3	0	0	0	0	0	0	0	0	0	0	5	0	5
Cellular Processes	Cell growth and death	Cell cycle - yeast	7	44	5	4	4	16	56	625	84	109	277	30	191	51	425	965	14	281	92	2733
Cellular Processes	Cell growth and death	Meiosis - yeast	84	1451	570	15	73	59	712	576	1202	95	629	58	1261	120	2876	1410	11	1792	118	4179
Cellular Processes	Cell growth and death	Oocyte meiosis	0	0	0	0	0	0	0	0	0	0	0	0	0	0	0	0	0	2	0	0
Organismal Systems	Circulatory system	Adrenergic signaling in cardiomyocytes	0	0	0	0	0	0	0	0	0	0	0	0	0	0	0	0	0	2	0	0

Class1 name	Class2 name	Class3 name	M24008	M24015	M24016	M24026	M24033	M24039	FKDN3	FKDN1	FKDN2	FKDN4	FKDN5	FKDN6	FKDN7	FKDN8	FKDN9	FKDN10	FKDN11	FKDNs1	FKDNs3	FKDNs4
Organismal Systems	Circulatory system	Vascular smooth muscle contraction	0	0	0	0	0	0	0	0	0	0	0	0	0	0	0	0	0	2	0	0
Environmental Information Processing	Signal transduction	Wnt signaling pathway	0	0	0	0	0	0	0	0	0	0	0	0	0	0	42	17	0	0	0	48
Environmental Information Processing	Signal transduction	Notch signaling pathway	0	0	0	0	0	0	0	0	0	0	0	0	0	0	42	17	0	0	0	48
Organismal Systems	Endocrine system	Renin-angiotensin system	85	1234	476	21	8	68	281	591	1474	202	833	69	2296	187	3434	2459	20	4311	199	7387
Organismal Systems	Immune system	RIG-I-like receptor signaling pathway	184	585	1515	40	35	6476	964	5284	5152	8666	404	300	999	556	940	3564	36	837	64	2406
Organismal Systems	Immune system	Toll and Imd signaling pathway	0	0	0	0	0	13	0	0	0	0	0	0	0	0	0	0	0	0	0	0
Organismal Systems	Immune system	Hematopoietic cell lineage	0	0	0	0	0	0	0	0	0	0	0	3	0	0	0	0	0	0	0	0
Organismal Systems	Immune system	Fc gamma R-mediated phagocytosis	0	2	0	0	0	0	0	3	0	0	0	0	0	0	0	0	0	0	0	0

Class1 name	Class2 name	Class3 name	M24 008	M24 015	M24 016	M24 026	M24 033	M24 039	FK DN 3	FK DN 1	FK DN 2	FK DN 4	FK DN 5	FK DN 6	FK DN 7	FK DN 8	FK DN 9	FK DN 10	FK DN 11	FK DN s1	FK DN s3	FK DN s4
Organismal Systems	Environmental adaptation	Circadian entrainment	0	0	0	0	0	0	0	0	0	0	0	0	0	0	0	0	0	2	0	0
Organismal Systems	Nervous system	Long-term potentiation	0	0	0	0	0	0	0	0	0	0	0	0	0	0	0	0	0	2	0	0
Organismal Systems	Nervous system	Neurotrophin signaling pathway	0	0	0	0	0	0	0	0	0	0	0	0	0	0	0	0	0	2	0	0
Organismal Systems	Nervous system	Serotonergic synapse	59	189	40	7	11	22	247	874	58	74	45	20	175	47	220	355	16	394	16	270
Organismal Systems	Nervous system	Dopaminergic synapse	59	189	40	7	11	22	247	874	58	74	45	23	175	47	220	355	16	396	16	270
Organismal Systems	Sensory system	Olfactory transduction	0	0	0	0	0	0	0	0	0	0	0	0	0	0	0	0	0	2	0	0
Organismal Systems	Sensory system	Phototransduction	0	0	0	0	0	0	0	0	0	0	0	0	0	0	0	0	0	2	0	0
Organismal Systems	Sensory system	Phototransduction - fly	0	0	0	0	0	0	0	0	0	0	0	0	0	0	0	0	0	2	0	0
Organismal Systems	Sensory system	Inflammatory mediator regulation of TRP channels	0	0	0	0	0	0	0	0	0	0	0	0	0	0	0	0	0	2	0	0
Organismal	Endocrine	GnRH signaling pathway	0	2	0	0	0	0	0	3	0	0	0	0	0	0	0	0	0	2	0	0

Class1 name	Class2 name	Class3 name	M24008	M24015	M24016	M24026	M24033	M24039	FKDN3	FKDN1	FKDN2	FKDN4	FKDN5	FKDN6	FKDN7	FKDN8	FKDN9	FKDN10	FKDN11	FKDNs1	FKDNs3	FKDNs4
Systems	system																					
Organismal Systems	Endocrine system	Melanogenesis	6	34	1	0	2	3	0	6	0	0	0	0	4	1	0	0	0	167	0	0
Organismal Systems	Endocrine system	Adipocytokine signaling pathway	19107	4676	2988	150	428	12737	6296	9401	10348	9484	12502	15329	6691	2005	12239	9272	123	8887	2104	12415
Organismal Systems	Endocrine system	Oxytocin signaling pathway	0	0	0	0	0	0	0	0	0	0	0	0	0	0	0	0	0	2	0	0
Organismal Systems	Endocrine system	Aldosterone synthesis and secretion	0	0	0	0	0	0	0	0	0	0	0	0	0	0	0	0	0	2	0	0
Organismal Systems	Excretory system	Endocrine and other factor-regulated calcium reabsorption	0	0	1	2	0	1	0	0	1	0	0	0	0	4	3	0	0	0	0	76
Organismal Systems	Digestive system	Gastric acid secretion	0	0	0	0	0	0	0	0	0	0	0	0	0	0	0	0	0	2	0	0
Organismal Systems	Digestive system	Protein digestion and absorption	369	157	210	10	10	32	157	273	509	98	273	33	1360	90	708	1145	12	3185	92	3474
Human Diseases	Substance dependence	Cocaine addiction	59	189	40	7	11	22	247	874	58	74	45	20	175	47	220	355	16	394	16	270
Human Diseases	Substance dependence	Amphetamine addiction	59	189	40	7	11	22	247	874	58	74	45	20	175	47	220	355	16	396	16	270

Class1 name	Class2 name	Class3 name	M24008	M24015	M24016	M24026	M24033	M24039	FKDN3	FKDN1	FKDN2	FKDN4	FKDN5	FKDN6	FKDN7	FKDN8	FKDN9	FKDN10	FKDN11	FKDNs1	FKDNs3	FKDNs4
Human Diseases	Infectious diseases: Bacterial	Bacterial invasion of epithelial cells	35710	3351	43	15793	28	17229	3721	18	257	690	1137	208	834	1634	264	818	13	563	444	2056
Human Diseases	Infectious diseases: Bacterial	Pathogenic Escherichia coli infection	4	0	0	0	0	2	0	0	0	0	0	0	0	0	0	0	0	0	0	0
Human Diseases	Infectious diseases: Bacterial	Shigellosis	0	0	0	0	0	264	6	8	77	388	212	105	242	1088	170	179	2	138	292	1330
Human Diseases	Infectious diseases: Bacterial	Staphylococcus aureus infection	5654	34028	11644	183958	10907	62911	30551	1659	5767	1135	20557	219	1703	84	4287	5758	166	1339	2741	6379
Human Diseases	Cancers: Specific types	Glioma	0	0	0	0	0	0	0	0	0	0	0	0	0	0	0	0	0	2	0	0
Human Diseases	Cancers: Specific types	Chronic myeloid leukemia	0	0	0	0	0	0	0	0	0	0	0	0	0	0	42	17	0	0	0	48
Human Diseases	Immune diseases	Systemic lupus erythematosus	3	68	12	0	2	1	2	465	61	25	10	4	17	18	1	4	4	74	0	30

Class1 name	Class2 name	Class3 name	M24008	M24015	M24016	M24026	M24033	M24039	FKDN3	FKDN1	FKDN2	FKDN4	FKDN5	FKDN6	FKDN7	FKDN8	FKDN9	FKDN10	FKDN11	FKDNs1	FKDNs3	FKDNs4
Human Diseases	Cardiovascular diseases	Hypertrophic cardiomyopathy (HCM)	18	115	11	6	1	18	6	190	91	94	278	29	812	68	426	1185	8	1625	103	3224

Class 1 represents the name of the first-level classification level metabolic pathway; Class 2 represents the name of the second-level classification level metabolic pathway; Class 3 represents the name of the third-level classification level metabolic pathway. The higher the level, the more refined the metabolic pathway; M24008, M24015, M24016, etc. represent the copy number of the expressed genes for each metabolic pathway.

Table S13. FAPROTAX Ecosystem Function Prediction Results

Function	FKDN: mean rel.freq.(%)	M: mean rel.freq. (%)	FKDN: std.dev. (%)	M: std.dev. (%)	*p*-values	*p*-values (corrected)	Difference between means	95.0% lower CI	95.0% upper CI
methanotrophy	0.001810457	0	0.006527696	0	0.335561278	0.650785509	0.001810457	-0.002100798	0.005721712
sulfate respiration	0	0.000776485	0	0.001627973	0.334953356	0.691516606	-0.000776485	-0.002648001	0.001095031
respiration of sulfur compounds	0	0.000776485	0	0.001627973	0.334953356	0.669906712	-0.000776485	-0.002648001	0.001095031
arsenite oxidation detoxification	0.022733271	0.008087046	0.033017963	0.018083185	0.248102907	0.635143442	0.014646225	-0.011259175	0.040551624
dissimilatory arsenite oxidation	0.022733271	0.008087046	0.033017963	0.018083185	0.248102907	0.610714848	0.014646225	-0.011259175	0.040551624
nitrate denitrification	0.151298319	0.028084263	0.231560474	0.039700401	0.084456253	0.386085727	0.123214055	-0.01894473	0.265372841
nitrite denitrification	0.163118653	0.028084263	0.230556991	0.039700401	0.060132331	0.384846921	0.13503439	-0.006539995	0.276608774
nitrous oxide denitrification	0.163118653	0.028084263	0.230556991	0.039700401	0.060132331	0.349860837	0.13503439	-0.006539995	0.276608774
denitrification	0.163118653	0.028084	0.23055	0.03970	0.060132	0.32070576	0.1350343	-0.0065399	0.2766087

Function	FKDN: mean rel.freq.(%)	M: mean rel.freq. (%)	FKDN: std.dev. (%)	M: std.dev. (%)	*p*-values	*p*-values (corrected)	Difference between means	95.0% lower CI	95.0% upper CI
		263	6991	0401	331	8	9	95	74
chitinolysis	0.95615407	0.001686737	1.196576276	0.003208266	0.012992399	0.415756769	0.954467333	0.237500628	1.671434038
knallgas bacteria	0.001810435	0	0.002863962	0	0.040176551	0.367328467	0.001810435	9.44E-05	0.003526459
nitrite ammonification	0.527617167	2.708838564	0.970221341	2.684727268	0.131031289	0.465889027	-2.181221396	-5.258151478	0.895708686
nitrite respiration	0.690735821	2.736922827	0.996601021	2.662542562	0.149199323	0.50256614	-2.046187006	-5.097452123	1.005078111
dark sulfide oxidation	0.012300241	0	0.022042176	0	0.065416645	0.322051174	0.012300241	-0.000906956	0.025507438
dark sulfur oxidation	0.011617918	0	0.04020317	0	0.316433022	0.675057114	0.011617918	-0.012470956	0.035706791
invertebrate parasites	1.679508785	0	3.726077557	0	0.128109425	0.482294305	1.679508785	-0.553076622	3.912094193
human pathogens septicemia	3.979553336	0.988983972	2.953455918	1.924362415	0.024841897	0.317976285	2.990569364	0.438934333	5.542204395
human pathogens pneumonia	4.001842552	1.002662398	2.95695351	1.919541782	0.024358357	0.38973371	2.999180154	0.450299018	5.54806129

Function	FKDN: mean rel.freq.(%)	M: mean rel.freq. (%)	FKDN: std.dev. (%)	M: std.dev. (%)	*p*-values	*p*-values (corrected)	Difference between means	95.0% lower CI	95.0% upper CI
human pathogens nosocomia	3.995873958	1.015318179	2.961584789	1.913925547	0.024950846	0.26614236	2.980555779	0.43467657	5.526434987
human pathogens meningitis	0.291990852	2.839489451	0.577830068	3.657998655	0.180782025	0.525911345	-2.5474986	-6.748971131	1.653973932
human pathogens gastroenteritis	1.36712322	3.304182751	1.186450679	2.296932467	0.122214867	0.488859469	-1.93705953	-4.570663403	0.696544342
human pathogens diarrhea	0.72951557	3.307854457	1.037707778	2.286707291	0.052711492	0.421691934	-2.578338888	-5.19868979	0.042012015
plant pathogen	1.044076778	0.032162923	1.20489237	0.048688911	0.009741021	0.623425327	1.011913855	0.289054	1.73477371
aromatic compound degradation	0.951281878	0.044288142	1.187783892	0.063327915	0.016574018	0.353579048	0.906993736	0.193724034	1.620263438
aliphatic non methane hydrocarbon degradation	0.005504128	0	0.019845416	0	0.335561278	0.631644758	0.005504128	-0.006386818	0.017395074
dark iron oxidation	0.000479906	0	0.001175556	0	0.164833348	0.527466714	0.000479906	-0.000224461	0.001184274
iron respiration	0.01374413	0.000843851	0.03759334	0.001603936	0.238725498	0.636601328	0.012900279	-0.009656697	0.035457255
fumarate respiration	0.793220724	3.300030	1.02499	2.29430	0.058283	0.41446101	-2.5068096	-5.1358012	0.1221819

**Function**	**FKDN: mean rel.freq.(%)**	**M: mean rel.freq. (%)**	**FKDN: std.dev. (%)**	**M: std.dev. (%)**	***p*-values**	***p*-values (corrected)**	**Difference between means**	**95.0% lower CI**	**95.0% upper CI**
		35	8693	7376	579		26	22	71
intracellular parasites	0.004638013	2.06E-05	0.015143282	4.62E-05	0.29155211	0.643425347	0.004617364	-0.00445623	0.013690957
chlorate reducers	0.000479906	0	0.001175556	0	0.164833348	0.502349252	0.000479906	-0.000224461	0.001184274

Function represents the name of the ecological function; FKDN:mean rel.freq. (%) represents the proportion of genes expressing a certain ecological function in the total sequenced fragments in the Tazhong Town group. M:mean rel.freq. (%) represents the proportion of genes expressing a certain ecological function in the total sequenced fragments in the Pamir Plateau group. FKDN:std.dev. (%) represents the fluctuation range of the expression level of a single sample in the Tazhong Town group compared to the average level of the population. If the standard deviation of the expression level of a certain gene in multiple samples is large, it indicates that the expression level varies significantly among different samples; M:std.dev. (%) represents the fluctuation range of the expression level of a single sample in the Pamir Plateau group compared to the average level of the population; *p* values represent the original *p* values obtained through hypothesis testing (such as t-test, etc.), if the *p* value is less than 0.05, it indicates a significant difference; *p* values (corrected): the corrected values of the original *p* values to control the false positive rate and to more accurately determine the significance of the differences between groups; Difference between means: the difference in the average relative proportion of the corresponding functional genes between the FKDN group and the M group, which can directly understand the average level difference of the two groups in this function; 95.0% lower CI: the lower limit of the 95% confidence interval of the difference in the average relative proportion of the corresponding functional genes, indicating that the true difference may be greater than or equal to this lower limit value; 95.0% upper CI: the upper limit of the 95% confidence interval of the difference in the average relative level of the corresponding functional genes, meaning that the true difference may be less than or equal to this upper limit value.
